# A comparison of methods to generate adaptive reference ranges in longitudinal monitoring

**DOI:** 10.1371/journal.pone.0247338

**Published:** 2021-02-19

**Authors:** Davood Roshan, John Ferguson, Charles R. Pedlar, Andrew Simpkin, William Wyns, Frank Sullivan, John Newell

**Affiliations:** 1 School of Mathematics, Statistics and Applied Mathematics, National University of Ireland Galway, Galway, Ireland; 2 CÚRAM, SFI Research Centre for Medical Devices, National University of Ireland, Galway, Ireland; 3 HRB Clinical Research Facility, National University of Ireland Galway, Galway, Ireland; 4 Faculty of Sport, Health and Applied Science, St Mary’s University, Twickenham, United Kingdom; 5 The Lambe Institute for Translational Medicine, National University of Ireland, Galway, Ireland; 6 Prostate Cancer Institute, National University of Ireland Galway, Galway, Ireland; Sichuan University, CHINA

## Abstract

In a clinical setting, biomarkers are typically measured and evaluated as biological indicators of a physiological state. Population based reference ranges, known as ‘static’ or ‘normal’ reference ranges, are often used as a tool to classify a biomarker value for an individual as typical or atypical. However, these ranges may not be informative to a particular individual when considering changes in a biomarker over time since each observation is assessed in isolation and against the same reference limits. To allow early detection of unusual physiological changes, adaptation of static reference ranges is required that incorporates within-individual variability of biomarkers arising from longitudinal monitoring in addition to between-individual variability. To overcome this issue, methods for generating individualised reference ranges are proposed within a Bayesian framework which adapts successively whenever a new measurement is recorded for the individual. This new Bayesian approach also allows the within-individual variability to differ for each individual, compared to other less flexible approaches. However, the Bayesian approach usually comes with a high computational cost, especially for individuals with a large number of observations, that diminishes its applicability. This difficulty suggests that a computational approximation may be required. Thus, methods for generating individualised adaptive ranges by the use of a time-efficient approximate Expectation-Maximisation (EM) algorithm will be presented which relies only on a few sufficient statistics at the individual level.

## 1 Introduction

Biomarkers are valuable potential indicators of disease prognosis. They play a crucial role in understanding the underlying pathogenesis of disease and extending knowledge of what is considered normal, healthy physiology. Static reference intervals are usually established to explain the variability of biomarkers in a ‘healthy’ sample of individuals and are widely used as a decision tool to classify individuals into normal/abnormal health status. In particular, a reference interval is estimated using a sample of data where the aim is to provide a range that will contain a pre-specified proportion (often 95%) of the underlying distribution or population with a certain degree of confidence [1]. These estimated reference ranges are referred to as ‘Tolerance Intervals‘. More formally, a (100*p, 100*(1 − *α*))% two-sided tolerance interval of the form (*L*(**Y**), *U*(**Y**)) which covers at least a proportion of *p* of the population with a confidence level of (1 − *α*) should satisfy:

$$\begin{array}{c} P\{F[U(Y)]-F[L(Y)]\geq p\}=1-\alpha \end{array}$$ (1)

where **Y** = (*Y*_1_, *Y*_2_, …, *Y*_*n*_) is random sample from a continuous random variable with the cumulative distribution function *F*.

In practice, it is crucial to estimate reference intervals appropriately. For example, the clinical and biological assessment of individuals is usually based on longitudinal monitoring of their biomarkers. Tolerance (static) reference intervals, while very useful when there is only one measurement available for an individual, are not reflective of a particular individual when biomarkers are collected longitudinally (i.e. multiple measurements per individual). Therefore, reference ranges which adapt to account for both between and within subject variabilities are needed for effective monitoring.

For example, in longitudinal monitoring programs in elite sports it is of interest to identify abnormal biological values in athletes’ blood biomarkers. One approach involves computing a Z-score based on unbiased estimates of the mean and variance of the previous observations. Letting *Y*_1_, …, *Y*_*n*_ be a sample of *n* independent random variables with *Y*_*i*_ ∼ *N*(*μ*_*i*_, *σ*^2^), observation *y*_*n*+1_ will be detected as abnormal by comparing the observed statistics |*z*_*n*+1_| in (2)

$$\begin{array}{c} Z_{n+1}=\frac{y_{n+1}-{\bar{Y}}_{n}}{{\hat{\sigma }}_{n}\sqrt{1+\frac{1}{n+1}}} \end{array}$$ (2)

with the $1-\frac{\alpha }{2}$ quantile of the standard normal distribution; where, ${\bar{Y}}_{n}$ and ${\hat{\sigma }}_{n}$ are the empirical mean and variance of the previous observations. Sottas et. al. remarked that the proposed Z-score is not suitable for small sample sizes as the distribution of the statistics in (2) is not well approximated by a *N*(0, 1) [2]. However, in a recent paper published by Sauliere et. al. the exact distribution was shown to be the Student *t* distribution with *n* − 1 degrees of freedom under the null hypothesis *H*_0_: *μ*_1_ = *μ*_2_ = … = *μ*_*n*+1_ = *μ* [3]. In their paper, three Z-score approaches were introduced to detect abnormal observations in a sample of an individual’s hematological markers. Their methods rely on the individual level observations only, indicating that some sensitivity or specificity might be lost when compared to a method that incorporates data from other individuals [2].

Sharpe et. al. proposed an alternative approach in which they include population-based information in the calculation of the Z-score (3) which can be applied to samples of arbitrary size [4]. In their approach, a universal within-subject variability (${\hat{\sigma }}_{uni}$), fixed for all subjects, was estimated.

$$\begin{array}{c} Z_{n+1}=\frac{x_{n+1}-{\bar{X}}_{n}}{{\hat{\sigma }}_{uni}\sqrt{1+\frac{1}{n+1}}} \end{array}$$ (3)

This assumption can cause a significant loss in sensitivity if a subject has a variance smaller than ${\hat{\sigma }}_{uni}^{2}$ [2].

Bayesian approaches provide a useful framework to incorporate information from the general population with the measurements for a given individual to investigate whether a new observation can be deemed as atypical. This approach for generating reference ranges was implemented by Sottas et. al. to detect athletes with abnormal profile for certain steroids [2]. The use of Bayesian techniques in detecting abnormal values was further studied in a wide range of applications by Sottas et. al., Robinson et. al., Pottgiesser et. al. and Lobigs et. al. [5–17]. In addition to these, the Parametric Empirical Bayes (PEB) approach proposed by McIntosh and Urban [18] and McIntosh et. al. [19] has been applied for screening ovarian cancer as an alternative to the single-threshold (ST) rule (or reference intervals) which counts subjects as ‘positive’ when their biomarker value exceeds a predefined threshold. They have shown that the PEB algorithm outperforms the ST rule when high population heterogeneity exists in biomarker values. However, similar to the Sharpe approach, a shortcoming of all these models is the assumption that within-subject variability is equal for all subjects.

In this article, we develop and extend the current Bayesian approaches to generate dynamic personalised reference ranges for longitudinal monitoring of clinical biomarkers to accommodate differences in within-individual variability. This approach is more realistic, and will hopefully result in wider applicability. However, the computational burden of a fully Bayesian approach may diminish its applicability, especially in large datasets. Therefore, a new efficient method will also be proposed using the Expectation-Maximization (EM) algorithm which may allow rapid construction of longitudinal reference ranges in large datasets. We compare the benefits and drawbacks of both approaches in a comprehensive simulation study and will provide some real data applications.

The rest of the article is outlined as follows. In Section 2, dynamic reference ranges will be discussed using a fully Bayesian approach while in Section 3 we will propose a computationally efficient alternative using an approximate EM algorithm. In Section 4 the simulation study will be presented to compare and evaluate the proposed methods from the contexts of accuracy and speed. In Section 5, the proposed models are applied to biomarkers collected longitudinally in a clinical setting and amongst elite athletes. In the final section, we present our conclusions and suggest some extensions that could be implemented in the future.

## 2 Dynamic reference ranges using Bayesian approaches

Consider *I* independent individuals, with individual *i* consisting of *n*_*i*_ independent normally distributed measurements, *y*_*ij*_; *j* = 1, …, *n*_*i*_, each with an unknown mean *μ*_*i*_ and an unknown variance $\sigma_{i}^{2}$; that is:

$$\begin{array}{c} y_{ij}|\mu_{i},\sigma_{i}^{2}∼N\left(\mu_{i},\sigma_{i}^{2}\right),\;\;\;\text{for}\;j=1,\ldots ,n_{i};\;i=1,\ldots ,I \end{array}$$ (4)

where it is assumed that clinical biomarkers are either normally (or log-normally) distributed. It is reasonable to expect that *μ*_*i*_, which represents individual *i*’s mean, follows a shared distribution from which they are sampled independently. Therefore, a normal distribution with hyperparameters (*μ*, *τ*^2^) is plausible as a prior distribution for the parameter *μ*_*i*_, for the convenience of conjugacy as follows:

$$\begin{array}{c} \mu_{i}|\mu ,\tau^{2}∼N\left(\mu ,\tau^{2}\right)\;\;\;\text{for}\;i=1,\ldots ,I \end{array}$$ (5)

A weakly informative hyperprior will be assigned to *μ* as:

$$\begin{array}{c} \mu |\nu ∼N(0,\nu^{2}) \end{array}$$ (6)

where, *ν* is a constant, and assumed to be relatively large to cover a wide range of possible values for *μ*. Vague priors (e.g. Inverse Gamma (IG)) will be assumed for both the within and between subject variabilities as follows:

$$\begin{array}{l} \sigma_{i}^{2}|\alpha_{1},\beta_{1}\sim IG(\alpha_{1},\beta_{1});\;i=1,\ldots ,I\\ \tau^{2}|\alpha_{2},\beta_{2}\sim IG(\alpha_{2},\beta_{2}) \end{array}$$ (7)

where *α*_*i*_ = *β*_*i*_; *i* = 1, 2 are set to a common low value (e.g. 0.01, 0.001). The inverse gamma distributions are conditionally conjugative, meaning that the conditional posterior distribution of $\sigma_{i}^{2}$ and *τ*^2^ given other parameters is also inverse gamma. The joint posterior distribution of all parameters can then be written by combining the observed values, *y*_*ij*_, with the prior distribution as follows:

$$\begin{array}{c} \begin{array}{cc} p(\underset{˜}{\mu },\;{\underset{˜}{\sigma }}^{2},\mu ,\tau^{2}|Y) & \propto p\left(\tau^{2}\right)p\left(\mu \right)\prod_{i=1}^{I}p\left(\mu_{i}|\mu ,\tau^{2}\right)p\left(\sigma_{i}^{2}\right)\prod_{i=1}^{I}\prod_{j=1}^{n_{i}}p\left(y_{ij}|\mu_{i},\sigma_{i}^{2}\right)\\ & \propto IG\left(\tau^{2}|\alpha_{2},\beta_{2}\right)N\left(\mu |0,\nu^{2}\right)\prod_{i=1}^{I}N\left(\mu_{i}|\mu ,\tau^{2}\right)IG\left(\sigma_{i}^{2}|\alpha_{1},\beta_{1}\right)\prod_{i=1}^{I}\prod_{j=1}^{n_{i}}N\left(y_{ij}|\mu_{i},\sigma_{i}^{2}\right) \end{array} \end{array}$$ (8)

where, $\underset{˜}{\mu }=(\mu_{1},\ldots ,\mu_{I})$ and ${\underset{˜}{\sigma }}^{2}=\left(\sigma_{1}^{2},\ldots ,\sigma_{I}^{2}\right)$ are the vectors of individual means and variances respectively, and *Y* refers to the observed values of *y*_*ij*_; *i* = 1, …, *I* & *j* = 1, …, *n*_*i*_. Consequently, the posterior predictive distribution of $P(y_{i(n_{i}+1)}|Y)$ can be obtained as:

$$\begin{array}{c} p\left(y_{i(n_{i}+1)}|Y\right)=\int \ldots \int p\left(y_{i(n_{i}+1)}|\mu_{i},\sigma_{i}^{2}\right)p\left(\theta |Y\right)d\theta \end{array}$$ (9)

where ***θ*** = ($\underset{˜}{\mu }$, ${\underset{˜}{\sigma }}^{2}$, *μ*, *τ*^2^) is a vector consisting of all parameters and hyperparameters. Future measurement (*n*_*i*_ + 1) from subject *i*, $y_{i(n_{i}+1)}$, will be considered atypical if it falls outside the $\frac{\alpha }{2}*100\%$ and $(1-\frac{\alpha }{2})*100\%$ quantiles of $P(y_{i(n_{i}+1)}|Y)$.

The integrals in (8) and (9) needed to make inferences are not tractable analytically and have no closed form and hence must be approximated through a numerical approach. A Gibbs sampler was implemented, as the full conditional posterior distributions have closed forms and can be sampled from as follows:

$$\begin{array}{c} p\left(\mu |\tau^{2},{\underset{˜}{\sigma }}^{2},\underset{˜}{\mu },Y\right)∼N(\frac{\frac{\sum_{i=1}^{I}\mu_{i}}{\tau^{2}}}{\frac{1}{\nu^{2}}+\frac{I}{\tau^{2}}}\;,\;\frac{1}{\frac{1}{\nu^{2}}+\frac{I}{\tau^{2}}}) \end{array}$$ (10)

$$\begin{array}{c} \begin{array}{cc} p(\tau^{2}|\mu ,{\underset{˜}{\sigma }}^{2},\underset{˜}{\mu },Y) & ∼IG(\alpha_{2}+\frac{I}{2}\;,\;\beta_{2}+\frac{1}{2}\sum_{i=1}^{I}{\left(\mu_{i}-\mu \right)}^{2}) \end{array} \end{array}$$ (11)

$$\begin{array}{c} \begin{array}{cc} p(\sigma_{i}^{2}|\mu ,\tau^{2},{\underset{∼}{\sigma }}_{-i}^{2},\underset{˜}{\mu },Y) & ∼IG(\alpha_{1}+\frac{n_{i}}{2}\;,\;\beta_{1}+\frac{1}{2}\sum_{j=1}^{n_{i}}{\left(y_{ij}-\mu_{i}\right)}^{2}) \end{array} \end{array}$$ (12)

$$\begin{array}{c} \begin{array}{cc} p(\mu_{i}|\tau^{2},{\underset{˜}{\sigma }}^{2},{\underset{∼}{\mu }}_{-i},Y) & ∼N(\frac{\frac{\mu }{\tau^{2}}+\frac{\sum_{j=1}^{n_{i}}y_{ij}}{\sigma_{i}^{2}}}{\frac{1}{\tau^{2}}+\frac{n_{i}}{\sigma_{i}^{2}}}\;,\;\frac{1}{\frac{1}{\tau^{2}}+\frac{n_{i}}{\sigma_{i}^{2}}}) \end{array} \end{array}$$ (13)

The Gibbs sampler will draw samples alternately by sampling from (10)–(13) as appropriate. Once the samples are drawn, the posterior predictive simulations of new data, $y_{i(n_{i}+1)}$, can be obtained by sampling from (4) given the generated (***θ***_**1**_, …, ***θ***_***t***_). Finally, the reference range will be defined as the ‘middle’ (1 − *α*)% quantiles of the predicted $y_{i(n_{i}+1)}\text{s}$.

Since estimation of the within-subject variabilities required at least two measurements, using information gleaned from the population allows the construction of reference ranges for the first two measurements of an individual using (static) reference ranges. These ranges are then adapted as more observations are gathered on the same subject using the Bayesian approach. These reference ranges will be updated based on that individual’s records, and less based on the records for other individuals in the database, as the number of biomarker values becomes very large for the individual.

## 3 Dynamic reference ranges using approximate Expectation Maximization algorithm

The Bayesian approach proposed above is computationally intensive. This is problematic for large data streaming problems (i.e. where data are gathered continuously at a high rate) since the model parameters need to be re-estimated using Markov chain Monte Carlo (MCMC) whenever new data are gathered. For example, these days large amounts of data are collected rapidly using glucose sensors to continuously monitor glucose levels in diabetic patients. Therefore, in order to make the algorithm faster and memory efficient, the procedure of developing the proposed ranges could be based on some sufficient statistics from the previous data points rather than relying on the entire current dataset [20]. In this regard, a random intercept model will be defined for the biomarkers’ values as:

$$\begin{array}{c} y_{ij}=\mu_{i}+\epsilon_{ij},\;\;\;\;i=1,\ldots ,I,\;j=i,\ldots ,n_{i} \end{array}$$ (14)

where *y*_*ij*_ represents the *j*’th biomarker value for subject *i* and is assumed to be normally distributed as $y_{ij}∼N\left(\mu_{i},\sigma_{i}^{2}\right)$. The *μ*_*i*_s are the subject level random intercepts that are also assumed to have a normal distribution as *μ*_*i*_ ∼ *N*(*μ*, *τ*^2^). Finally the error terms indicated by *ϵ*_*ij*_ are assumed to be independent of *μ*_*i*_ and normally distributed, i.e. $\epsilon_{ij}∼N\left(0,\sigma_{i}^{2}\right)$. For convenience, we also set $\gamma_{i}=\left(\mu ,\tau^{2},\sigma_{i}^{2}\right)$.

Measurement *j* from subject *i*, *y*_*ij*_, will be considered as ‘atypical’ if it falls beyond the $\frac{\alpha }{2}*100\%$ and $(1-\frac{\alpha }{2})*100\%$ quantiles of the distribution of *P*(*y*_*ij*_|*y*, *γ*_*i*_); where *y* refers to the both subject *i* measurements prior to time *j* (i.e. *y*_*i*1_, …, *y*_*ij*−1_) and other historical information from other individuals (i.e. *y*_*i*′*j*_; *i*′ ≠ *i* & *j* = 1, …, *n*_*i*′_). Due to the (assumed) independence of individuals, *P*(*y*_*ij*_|*y*, *γ*_*i*_) can be written in the form $P(y_{ij}|{\bar{y}}_{i}^{j-1},\gamma_{i})$; where ${\bar{y}}_{i}^{j-1}$ is the average value of the biomarker measurements for subject *i* before time *j* and is assumed normally distributed as ${\bar{y}}_{i}^{j-1}|\mu_{i},\sigma_{i}^{2}∼N\left(\mu_{i},\frac{\sigma_{i}^{2}}{j-1}\right)$. Hence, the distribution of $P(y_{ij}|{\bar{y}}_{i}^{j-1},\gamma_{i})$ can be found through (15) as:

$$\begin{array}{c} \begin{array}{c} y_{ij}|{\bar{y}}_{i}^{j-1},\gamma_{i}∼N(\frac{\frac{\mu }{\tau^{2}}+\frac{\left(j-1\right){\bar{y}}_{i}^{j-1}}{\sigma_{i}^{2}}}{\frac{1}{\tau^{2}}+\frac{j-1}{\sigma_{i}^{2}}}\;,\;\frac{1}{\frac{1}{\tau^{2}}+\frac{j-1}{\sigma_{i}^{2}}}+\sigma_{i}^{2}) \end{array} \end{array}$$ (15)

where:

$$\begin{array}{c} \begin{array}{c} p\left(\mu_{i}|{\bar{y}}_{i}^{j-1},\gamma_{i}\right)∼N(\frac{\frac{\mu }{\tau^{2}}+\frac{\left(j-1\right){\bar{y}}_{i}^{j-1}}{\sigma_{i}^{2}}}{\frac{1}{\tau^{2}}+\frac{j-1}{\sigma_{i}^{2}}}\;,\;\frac{1}{\frac{1}{\tau^{2}}+\frac{j-1}{\sigma_{i}^{2}}}) \end{array} \end{array}$$ (16)

The unknown parameters of (15) and (16) (i.e. *γ*_*i*_) cannot be estimated directly using Maximum Likelihood as the latent variables *μ*_*i*_ *i* ≤ *I* are not observed. Therefore, the EM algorithm [21] will be used as an alternative approach to find the maximum likelihood estimates of the model parameters. Assuming the *μ*_*i*_s are known, the complete log-likelihood function of the random intercept model presented in (14) can be written in the form:

$$l(\gamma_{i}|y,\mu_{i})\propto -\frac{1}{2}\sum_{i=1}^{I}n_{i}ln\left(\sigma_{i}^{2}\right)-\frac{1}{2}\sum_{i=1}^{I}\sum_{j=1}^{n_{i}}\frac{{\left(y_{ij}-\mu_{i}\right)}^{2}}{\sigma_{i}^{2}}-\frac{I}{2}ln\left(\tau^{2}\right)-\frac{1}{2}\sum_{i=1}^{I}\frac{{\left(\mu_{i}-\mu \right)}^{2}}{\tau^{2}}$$ (17)

where $n=\sum_{i=1}^{I}n_{i}$ represent the total number of observations.

The E step of the algorithm calculates the conditional expectation of (17), given the previous estimates of the hyperparameters ${\hat{\gamma }}_{i(k-1)}=\left({\hat{\mu }}_{\left(k-1\right)},{\hat{\tau }}_{\left(k-1\right)}^{2},{\hat{\sigma }}_{i(k-1)}^{2}\right)$ where the indexing signifies estimates after running (*k* − 1) cycles of the algorithm. Through this process we derive new estimates:

$$\begin{array}{c} \begin{array}{c} {\hat{\mu }}_{i(k)}={\hat{\rho }}_{i(k)}{\bar{y}}_{i}+\left(1-{\hat{\rho }}_{i(k)}\right){\hat{\mu }}_{\left(k-1\right)} \end{array} \end{array}$$ (18)

and

$$\begin{array}{c} \begin{array}{c} {\hat{\nu }}_{i(k)}={\hat{\tau }}_{\left(k-1\right)}^{2}\left(1-{\hat{\rho }}_{i(k)}\right) \end{array} \end{array}$$ (19)

where ${\hat{\rho }}_{i(k)}$ equals:

$$\begin{array}{c} {\hat{\rho }}_{i(k)}=\frac{{\hat{\tau }}_{\left(k-1\right)}^{2}}{{\hat{\tau }}_{\left(k-1\right)}^{2}+\frac{{\hat{\sigma_{i}}}_{\left(k-1\right)}^{2}}{n_{i}}} \end{array}$$ (20)

and represents the extent that the estimated individual mean, ${\hat{\mu }}_{i}$, is moving toward the overall mean, $\hat{\mu }$. Finally, the new estimates of model parameters at iteration *k*, i.e. ${\hat{\gamma }}_{i(k)}$, can be obtained as:

$$\begin{array}{c} \begin{array}{c} {\hat{\mu }}_{\left(k\right)}=\frac{\sum_{i=1}^{I}{\hat{\mu }}_{i(k)}}{I} \end{array} \end{array}$$ (21)

$$\begin{array}{c} \begin{array}{c} {\hat{\tau }}_{\left(k\right)}^{2}=\frac{1}{I}\sum_{i=1}^{I}\left({\hat{\nu }}_{i(k)}+{\hat{\mu }}_{i(k)}^{2}\right)-{\hat{\mu }}_{k}^{2} \end{array} \end{array}$$ (22)

$$\begin{array}{c} \begin{array}{c} {\hat{\sigma }}_{i(k)}^{2}=\frac{1}{n_{i}}\sum_{j=1}^{n_{i}}[{\left(y_{ij}-{\hat{\mu }}_{i(k)}\right)}^{2}+{\hat{\nu }}_{i(k)}] \end{array} \end{array}$$ (23)

The calculation of (21), (22) and (23) represents the M step of the algorithm. Note that the estimation of *γ*_*i*_ in the M step can be derived from just three Complete Data Sufficient Statistics (CDSS) that are computed in the E step using the imputed values for the latent variable and the estimated model parameters from the previous step. These CDSSs are:

$$\begin{array}{c} T_{1(k)}=\sum_{i=1}^{I}{\hat{\mu }}_{i(k)} \end{array}$$ (24)

$$\begin{array}{c} T_{2(k)}=\sum_{i=1}^{I}\left({\hat{\nu }}_{i(k)}+{\hat{\mu }}_{i(k)}^{2}\right) \end{array}$$ (25)

$$\begin{array}{c} T_{3i(k)}=\sum_{j=1}^{n_{i}}[{\left(y_{ij}-{\hat{\mu }}_{i(k)}\right)}^{2}+{\hat{\nu }}_{i(k)}],\;\;\;i=1,\ldots ,I \end{array}$$ (26)

The M step can then be re-expressed as:

$$\begin{array}{c} {\hat{\mu }}_{\left(k\right)}=\frac{T_{1(k)}}{I} \end{array}$$ (27)

$$\begin{array}{c} {\hat{\tau }}_{\left(k\right)}^{2}=\frac{T_{2(k)}}{I}-{\hat{\mu }}_{\left(k\right)}^{2} \end{array}$$ (28)

$$\begin{array}{c} {\hat{\sigma }}_{i(k)}^{2}=\frac{T_{3i(k)}}{n_{i}},\;\;\;i=1,\ldots ,I \end{array}$$ (29)

In situations involving data streams or large datasets, the proposed EM algorithm becomes inefficient as the computation requires not only the complete data to be available at each iteration but also the entire estimation process to be repeated when a new data point is available. Ippel et. al. proposed an approximation of the Expectation Maximization (EM) algorithm to fit a random intercept model to large streaming datasets [22]. Unlike the EM algorithm, this new approach, which they call SEMA (Streaming EM Approximation), only stores summary statistics at the individual level rather than keeping all data in memory, and only updates the CDSS through the contribution for the individual for whom the new observation was entered. The E step can be approximated as below:

$$\begin{array}{c} T_{\left(t\right)}^{s}=T_{\left(t-1\right)}^{s}-T_{i_{t}\left(t-1\right)}^{s}+T_{i_{t}\left(t\right)}^{s} \end{array}$$ (30)

where *T*^*s*^ *s* are analogous to the CDSS described in (24), (25) and (26), except that they are now only approximate sufficient statistics. The iteration index which was denoted before by *k* is now replaced with *t* with *i*_*t*_ indicating the individual for whom the data were measured at time *t*. Also, $T_{i_{t}\left(t\right)}^{s}$ indicates the contribution to the CDSS for individual *i*_*t*_ at time *t*, and similarly $T_{i_{t}\left(t-1\right)}^{s}$ indicates the contribution to CDSS for individual *i*_*t*_ at time *t* − 1.

Unlike the standard EM algorithm, the contribution of other individuals whose values were not measured at time *t* remains the same as time *t* − 1, i.e. $T_{i(t)}^{s}=T_{i(t-1)}^{s}$ for *i* ≠ *i*_*t*_. To recalculate $T_{i_{t}}^{s}$, one needs only to calculate (18), (19) and (20) for subject *i*_*t*_.

In fact, these updates can be calculated using only ${\bar{y}}_{i}$, $\bar{y_{i}^{2}}$ and *n*_*i*_ indicating that storing the data is not required as only a few summaries are sufficient (See Ippel et. al. [22]). Once the approximate CDSSs are updated, the M step can be implemented analogously to (27), (28) and (29). Ippel et. al. have shown the SEMA performs almost as well as the normal EM algorithm when the dataset is sufficiently large and suggested combining the normal EM algorithm with SEMA in cases with small datasets [22].

Once the parameters are estimated by the SEMA procedure, the 100(1 − *α*)% dynamic reference ranges for subject *i* at time *j* will be generated by substituting the parameter estimates into (15) as follows:

$$\begin{array}{c} (\frac{\frac{\hat{\mu }}{{\hat{\tau }}^{2}}+\frac{(j-1){\bar{y}}_{i}^{j-1}}{{\hat{\sigma }}_{i}^{2}}}{\frac{1}{{\hat{\tau }}^{2}}+\frac{j-1}{{\hat{\sigma }}_{i}^{2}}}-z_{1-\frac{\alpha }{2}}\sqrt{\frac{1}{\frac{1}{{\hat{\tau }}^{2}}+\frac{j-1}{{\hat{\sigma }}_{i}^{2}}}+{\hat{\sigma }}_{i}^{2}} \end{array},\frac{\frac{\hat{\mu }}{{\hat{\tau }}^{2}}+\frac{(j-1){\bar{y}}_{i}^{j-1}}{{\hat{\sigma }}_{i}^{2}}}{\frac{1}{{\hat{\tau }}^{2}}+\frac{j-1}{{\hat{\sigma }}_{i}^{2}}}+z_{1-\frac{\alpha }{2}}\sqrt{\frac{1}{\frac{1}{{\hat{\tau }}^{2}}+\frac{j-1}{{\hat{\sigma }}_{i}^{2}}}+{\hat{\sigma }}_{i}^{2}})$$ (31)

Note the approach considered here can be considered a variant of Empirical Bayes where the streaming EM algorithm is used to estimate the hyperparameters of the model.

## 4 Simulation study

A simulation study was carried out to evaluate the performance of the proposed dynamic methods against the usual method to generate static reference ranges. The primary aim of the simulation study was to compare the capability of these approaches in detecting abnormal observations in a series of biomarker values at the individual level. For this purpose, the area under the Receiver Operating Characteristic (ROC) curve (AUC) will be measured to evaluate the diagnostic ability of these methods. Different scenarios were considered by varying the number of individuals in the population (I), the number of replicates per individual (*n*_*i*_), and the ratios of within-subject variabilities to between-subject variability ($r_{1}=\frac{Var(WSV)}{BSV}=\frac{Var(\sigma_{i}^{2})}{\tau^{2}}$ and $r_{2}=\frac{E(WSV)}{BSV}=\frac{E(\sigma_{i}^{2})}{\tau^{2}}$).

### 4.1 Simulation procedure

Data will be simulated from a normal distribution using $Y_{ij}∼N\left(\mu_{i},\sigma_{i}^{2}\right)$ for *i* = 1, …, *I* and *j* = 1, …, *n*_*i*_ as presented in (4) and according to the scenarios considered. The static and dynamic reference ranges will then be computed separately for each individual in a ‘leave-one-subject-out’ manner. In particular, if the reference ranges are required for subject *i*, the control cohort (i.e. the sample of healthy individuals) would contain all the *j* ≠ *i*, *j* = 1, …, *I* individuals for whom the static reference range will be developed using tolerance intervals presented in (1).

On the other hand, to examine the performance of dynamic reference ranges and compare them with static reference ranges in terms of detecting atypical observations, subject *i* will be randomly assigned to either a control (i.e. healthy) or case (i.e. diseased) subject. In particular, for a subject who is assigned to be a case, the last observation simulated will be altered according to:

$$\left\{ \begin{array}{cc} y_{in_{i}}^{test}=y_{in_{i}}-3\sigma_{i} & y_{in_{i}}<\mu_{i}\\ y_{in_{i}}^{test}=y_{in_{i}}+3\sigma_{i} & y_{in_{i}}\geq \mu_{i} \end{array}\right.$$

where $y_{in_{i}}^{test}=y_{in_{i}}$ for control subjects.

Since the estimation of model parameters, and as a result dynamic reference ranges, can be highly affected by abnormal observations in a series of observations, the last observation for cases is assumed to be atypical in order to ignore any bias in the estimated model parameters.

### 4.2 Simulation results

The results of the simulation study have shown that while all of the three models have comparable specificities, which are independent of the number of replications (*n*_*i*_), the Bayesian approach performed best in detecting abnormal observations with an overall AUC of 0.98 (Table 1).

**Table 1:** Simulation performance. The overall performance of the three methods for detecting abnormal observations.

Method	Average AUC
Static	0.88
Bayesian	0.98
Approximate EM	0.94

**Fig 1:**
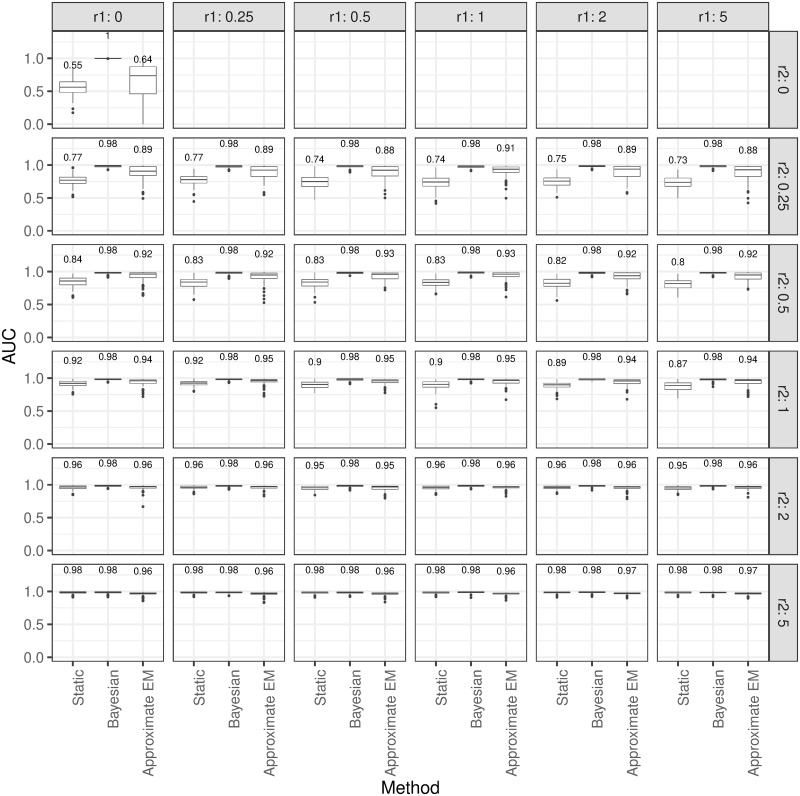
Performance of the static and dynamic reference ranges according to *r*_1_ and *r*_2_. The distribution of AUC for the three approaches (Static, Bayesian, and Approximate EM) in detection of outliers based on different combinations of *r*_1_ and *r*_2_.

The effect of *r*_1_ and *r*_2_ on the performance of the three different approaches has been displayed in the Fig 1 by comparing their boxplots. As shown, the overall performance of the Bayesian approach is the best for all combinations. However, when the WSV is large relative to the BSV (i.e., *r*_2_ is increasing), all three approaches resulted in similar performance suggesting the normal range is almost as good as the other two approaches. This result is to be expected since for large *r*_2_, individuals would have relatively the same behavior with a high homogeneity between them which suggests that deriving static reference ranges would not be much affected by a single individual in the sample. The performance of the three approaches remain relatively the same as *r*_1_ is changing which suggest *r*_1_ does not have a meaningful effect on the overall performance of the methods.

**Fig 2:**
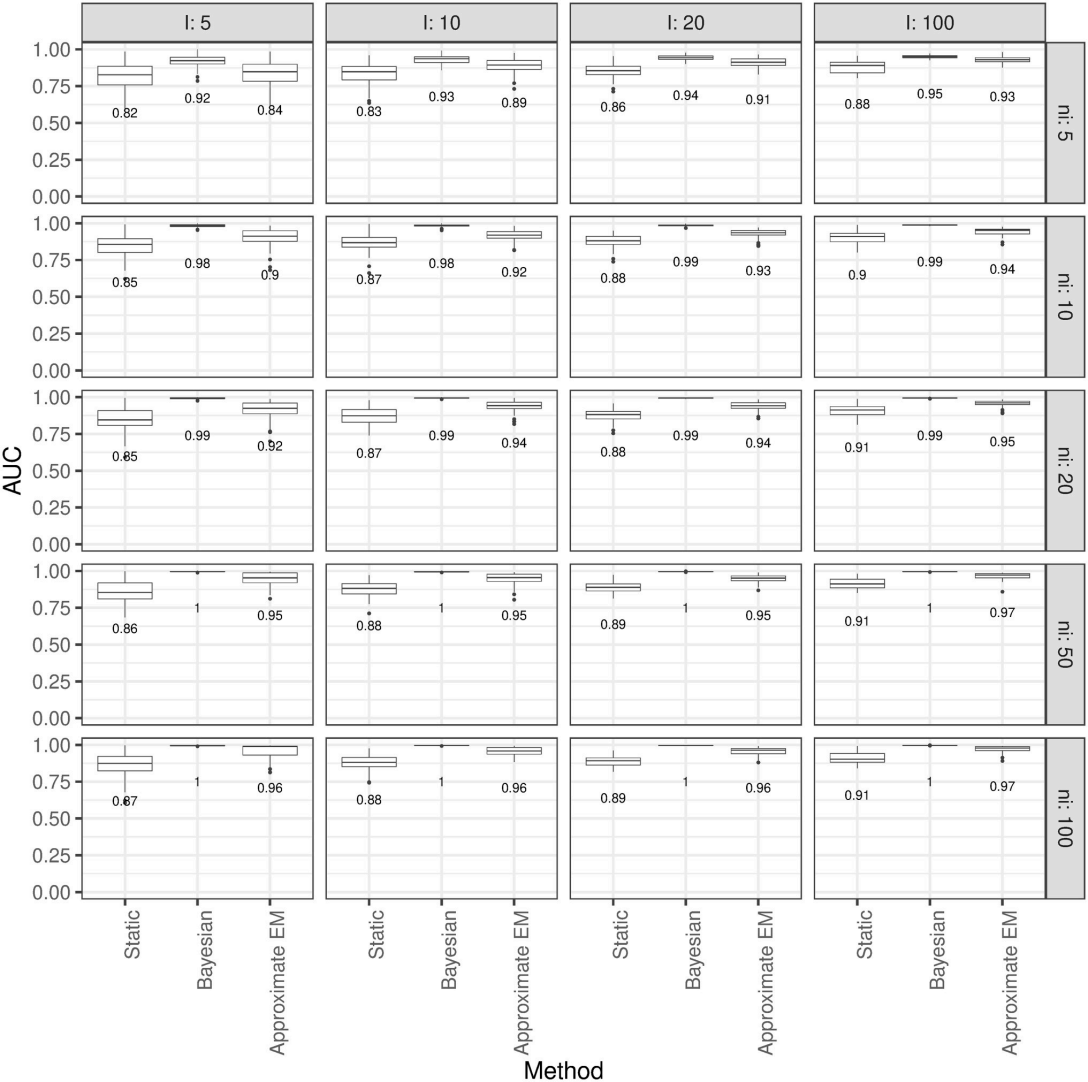
Performance of the static and dynamic reference ranges according to *I* and *n*_*i*_. The distribution of AUC for the three approaches (Static, Bayesian, and Approximate EM) in detection of outliers based on different combinations of *I* and *n*_*i*_.

The distributions of AUC for the three approaches based on different combinations of the number of individuals (*I*) and the number of replicates per individual, *n*_*i*_ has been also displayed in the Fig 2. In general, when both *I* and *n*_*i*_ increases, there is no noticeable difference between the Bayesian approach and the approximate EM algorithm with both outperforming the static method. Additionally, it can also be noticed from Fig 2 that the larger the number of individuals, the better the performance of the static method. However, the performance of the static reference ranges seems independent of *n*_*i*_. This might be expected as the static reference ranges are developed from a cohort of control subjects and as a result, the reference ranges for a specific subject is fixed and should be independent of his/her number of measurements.

In contrast, the results have shown when *n*_*i*_ increases there is a negligible difference between the Bayesian approach and the approximate EM algorithm. However, the approximate EM algorithm is considerably faster to implement compared to the Bayesian approach. Table 2 summarises the average time (in minutes) taken by each approach to generate reference ranges for each subject under different combinations of *I* and *n*_*i*_. It can be seen that there is a considerable difference between the Bayesian approach and the EM algorithm in their implementation time especially when the number of replications is large. For example in a worst-case scenario when there are 100 individuals (i.e., *I* = 100) for an individual with 100 measurements (i.e., *n*_*i*_ = 100), the Bayesian approach generates the reference ranges in about 75 minutes while it takes less than one minute to develop the reference ranges using the approximate EM algorithm. In terms of their performance, the average AUC for the Bayesian approach is 0.9960 while for the EM algorithm is 0.9702. Although there might be a small difference between the two approaches regarding their performance, the EM algorithm compensates for this in terms of implementation time. This trade-off between performance and time should be considered when implementing in practice.

**Table 2:** Simulation times. The average time (in minutes) taken by each approach to generate reference ranges baed on different combinations of *I* and *n*_*i*_.

I	Method	*n*_*i*_:5	*n*_*i*_:10	*n*_*i*_:20	*n*_*i*_:50	*n*_*i*_:100
5	Static	0.006	0.006	0.006	0.006	0.006
5	Bayesian	0.091	0.219	0.475	1.252	2.571
5	Approximate EM	0.012	0.013	0.016	0.031	0.081
10	Static	0.006	0.006	0.006	0.006	0.006
10	Bayesian	0.169	0.420	0.921	2.438	5
10	Approximate EM	0.019	0.020	0.023	0.038	0.089
20	Static	0.007	0.007	0.007	0.007	0.007
20	Bayesian	0.346	0.878	1.945	5.150	10.560
20	Approximate EM	0.033	0.034	0.037	0.053	0.104
100	Static	0.007	0.007	0.007	0.007	0.007
100	Bayesian	2.353	6.190	13.876	36.976	75.675
100	Approximate EM	0.140	0.141	0.145	0.188	0.314

## 5 Application

The findings from the simulation study gave an insight into the likely performance of the proposed methods and the parameters affecting their performance. In the remainder of this article, the use of dynamic reference ranges will be illustrated in clinical research and elite sport applications using longitudinal biomarker data. Subjects involved in these two studies consented to their data being used for research purposes. Ethics approval for longitudinal monitoring of glucose levels in diabetic patients was granted from the Galway University Hospital, National University of Ireland, Galway. Also, ethics approval for longitudinal monitoring of elite runners was granted from the Massachusetts General Hospital Institutional Review Board.

### 5.1 Longitudinal monitoring of glucose levels in diabetic patients

Diabetes is a metabolic disease that can cause various complications and even life-threatening consequences such as heart disease, kidney disease or stroke if not controlled appropriately [23]. In order to minimise the risk of these complications and hence control the progressive end-organ damage caused by diabetes, it is very important to keep blood sugar (Glucose) level within the healthy range. Both very high blood sugar (known as hyperglycemia) and very low blood sugar (hypoglycemia) lead to a number of symptoms. A Glucose level below 3.9 mmol/L [24] and above 11 mmol/L [25] are often considered as diagnostic range for people with diabetes. Therefore, continuous monitoring of glucose level is essential for the proper management of diabetes [23].

**Fig 3:**
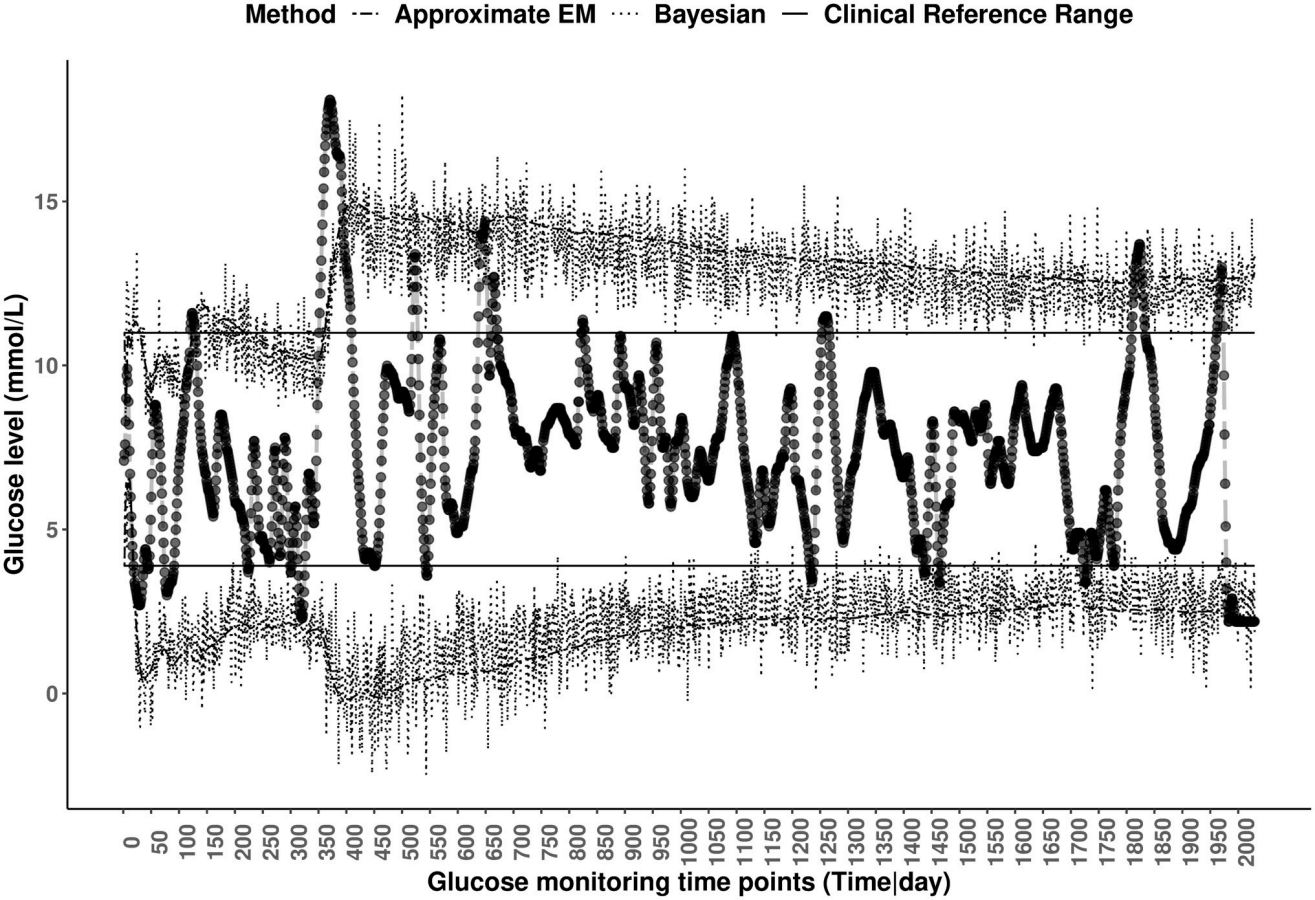
Static and dynamic reference ranges of glucose levels for a type 1 diabetic patient. A series of 2029 longitudinal glucose levels (longdashed line) for a type 1 diabetic patient with static reference ranges (solid lines), dynamic reference ranges using the Bayesian (dotted lines) and Approximate EM (dashed-dotted lines) methods.

The data considered here involved 18 type 1 diabetic adults where participants were fitted with a CGMS (iPro™ Professional Continuous Glucose Monitoring, Medtronic, USA) which captured the glucose measurements, and these were retrieved at the end of the observation period. The CGMS collected glucose readings in mmol/L every 10 minutes for 7 days, adding up to about 2016 glucose recordings per subject. The aim is to monitor the glucose levels of these patients by generating individualised dynamic reference ranges and compare them with the proposed clinical healthy range of [3.9, 11] mmol/L. Fig 3 displays dynamic reference ranges using both the Bayesian and approximate EM approaches along with the pre-defined clinical reference range for one such subjects.

As can be seen from Fig 3, the dynamic reference ranges are wider in general than the pre defined clinical reference range of [3.9, 11] mmol/L. The individual in question would be classified as hypoglycemic or hyperglycemic for all values outside the clinical reference ranges, while the dynamic reference range would classify several of these glucose values as non-hypoglycemic or non-hyperglycemic according to their own personal glucose trajectory. It is also observed that some glucose observations are not identified as atypical when using the (static) clinical reference range but have been identified as such when considering the dynamic reference ranges.

This information is summarised in Table 3 where the level of agreement between the clinical and approximate EM reference ranges is given. As opposed to pre-defined clinical reference range which weighs all subjects in the population equally, the proposed dynamic reference ranges are tailored to measurements observed on one subject, and as a result may better assists physicians in monitoring and assessing meaningful patterns in a patients’ glucose levels over time.

**Table 3:** Level of agreement. The level of agreement between the clinical and approximate EM reference ranges in classifying a sample patient’s glucose level as normal or abnormal.

		Clinical Reference Range		Total
		Abnormal	Normal	
Approximate EM	Abnormal	110	9	119
	Normal	178	1732	1910
	Total	288	1741	2029

It is worth noting that the computational time to generate the dynamic reference ranges was very much in favour of the approximate EM algorithm which took 1 minute as opposed to 1 hour for the full Bayesian implementation.

### 5.2 Longitudinal monitoring of elite runners

The measurement of haematological and biochemical variables are now widely used in elite sport to inform athlete training regimes. Monitoring these variables has the potential to help athletes avoid injury and illness via adjustments to diet and training load. For example, the measurement of hydroperoxides is an indirect measure of reactive intermediary by products of in-vivo lipid, protein, and nucleic acid oxidation (i.e. free radical activity) that increase with periods of excessive physical stress. These variables have been demonstrated to be sensitive to physiological stress, sleep loss and psychological stress in elite athletes, however individual physiological set points must be taken into account [26]. Elevated concentrations of hydroperoxides are associated with an increased risk of injury [27]. The data considered here comprises 11 elite distance runners of which four are male and seven are female. The aim is to identify meaningful changes in test results over their training period. Samples were collected in a standardised manner once per week, prior to exercise training. The approach taken is to construct static and dynamic reference ranges for each athlete to not only compare his/her test results with the rest of the athletes but also to see if there are any meaningful changes in his/her hydroperoxides.

**Fig 4:**
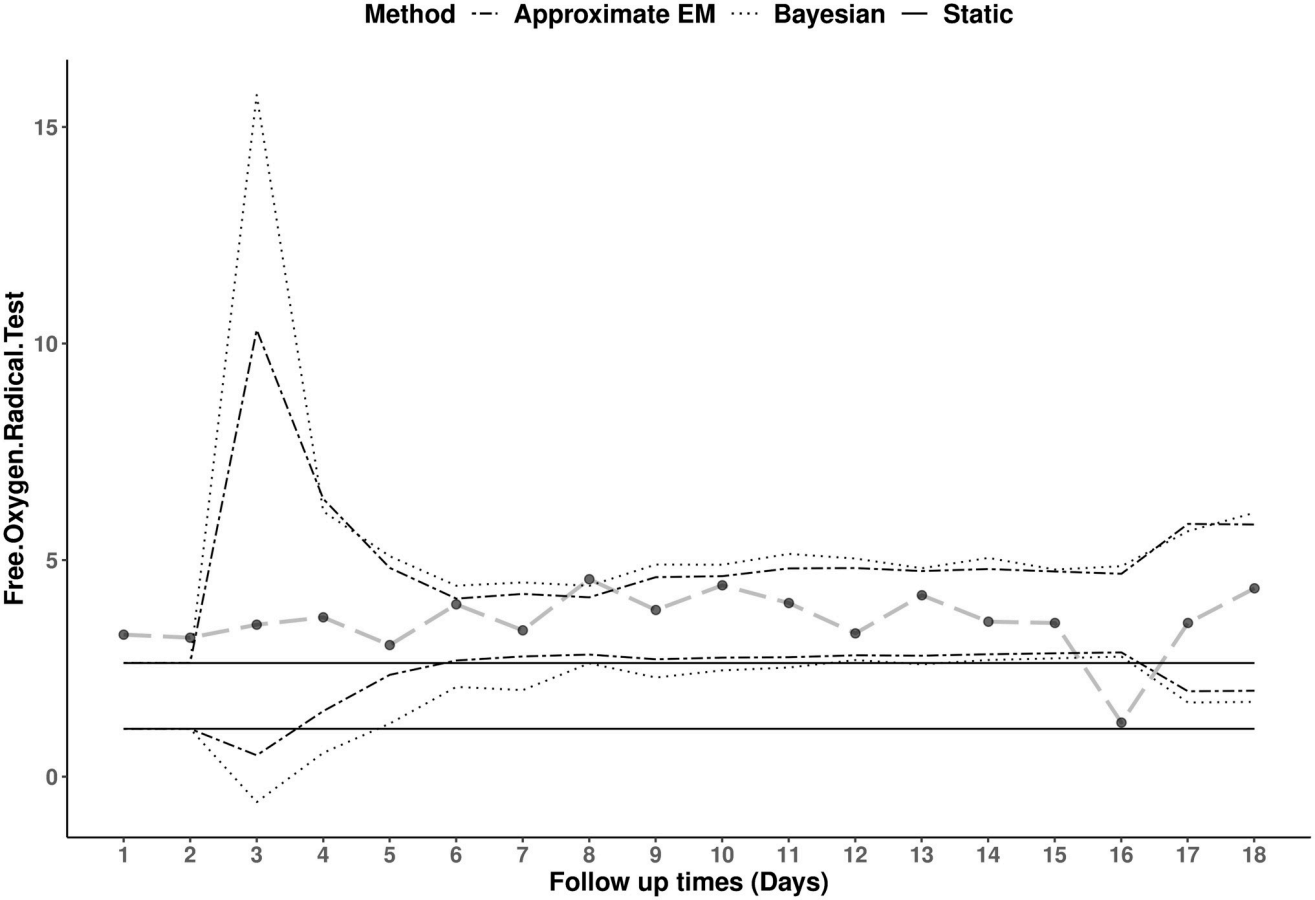
Static and dynamic reference ranges of free oxygen radical test for a female runner. A series of 18 longitudinal hydroperoxides values (longdashed line) for a female athlete with static reference ranges (solid lines), dynamic reference ranges using the Bayesian (dotted lines) and Approximate EM (dashed-dotted lines) methods.

**Fig 5:**
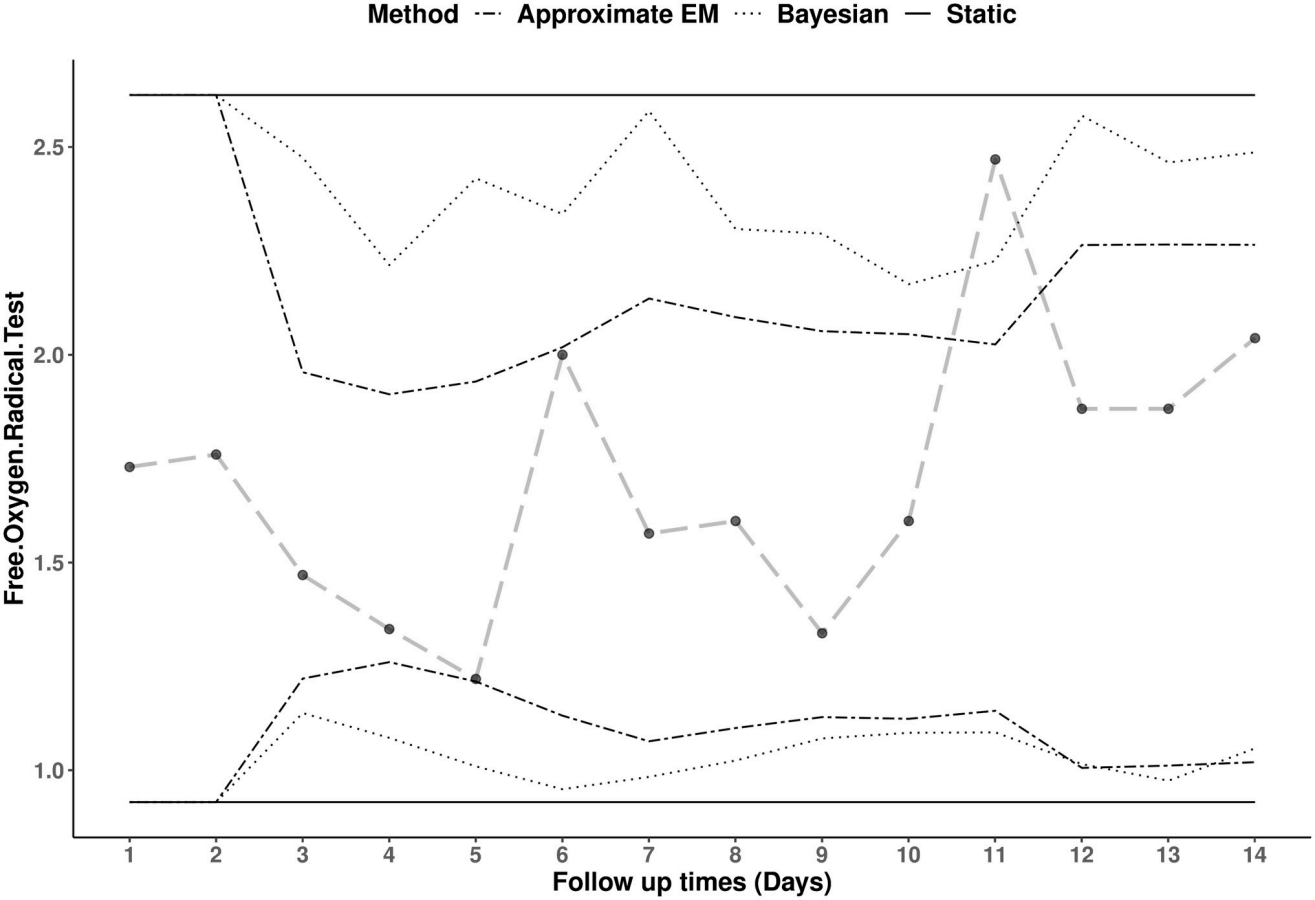
Static and dynamic reference ranges of free oxygen radical test for a male runner. A series of 14 longitudinal hydroperoxides values (longdashed line) for a male athlete with static reference ranges (solid lines), dynamic reference ranges using the Bayesian (dotted lines) and Approximate EM (dashed-dotted lines) methods.

According to the Harris and Boyd suggestions on stratifying the reference interval [28], data were split into two groups based on athletes’ gender, as the ratio of BSV within each gender exceeds 1.5. Log transformations were used to satisfy the normality assumption needed. Figs 4 and 5 show the reference ranges for two specific female and male athletes, respectively. Fig 4 reveals that, the female athlete in question has relatively higher hydroperoxides results compared to the rest of individuals in the sample and as a result the majority of her test results are outside the defined static reference ranges. This is an example where dynamic methods identified most of the test results as typical for that individual except for a considerable drop occurring at the 16^*th*^ follow up, while the value was considered as the only ‘usual’ value based on a static reference range.

On the other hand, as can be seen from Fig 5, the male athlete’s test results have relatively larger variability compared to the female athlete which results in similar reference ranges for all three approaches. Nevertheless, the dynamic reference ranges are still trying to adapt whenever a new observation is collected for the individual. This is why, the 11^*th*^ test result was found atypical for the subject while the static reference range treated that as a ‘typical’ observation.

## 6 Discussion

The use of statistical models to analyse longitudinal data is not limited to clinical setting or elite sports. There is an abundant research employing different algorithms for assessing serial observations or detection of disease onset [29]. Changepoint models are one of the popular algorithms used widely in this domain. Reference Ranges are also used in quality control, for example, Shewhart charts are one of the major tools in statistical process control to understand, monitor and control process performance. Cumulative Sum Control (CUSUM) charts are a useful and faster method to detect a small to moderate shift, in both the mean and variance of a process [30]. Regardless of the popularity of the proposed approaches in detecting a change point in a clinical setting or potential shifts in statistical process control, they are no suited to identification of ‘isolated abnormal’ values [31].

In this paper, an overview of methods for developing conventional (static) reference ranges for clinical biomarkers was presented and critiqued. The main concern in using population-derived reference ranges in practice is that biomarkers are typically collected longitudinally for subjects and this ‘serial’ component is often ignored. This is especially true when within-subject variability is considerably less than between-subject variability. This might cause a meaningful change in biomarker values for an individual to be missed by the use of static reference ranges. To overcome this, the use of Bayesian methods to generate dynamic reference ranges was recommended where combining an individual’s measurement with prior information gleaned from the population allows not only a more accurate quantification of the characteristics of the individual, but also to adapt and update the reference ranges as more data are collected for the individual. The advantage of Bayesian methods compare to other approaches (e.g. Z-score 2) in generating dynamic reference ranges was studied and highlighted in Sottas et. al. [2]. We further extended and compared the current Bayesian method by allowing different within-individual variability for each individual. In particular, our approach is almost identical to Sottas’ approach in the case where within subject variability is constant, and so the comparisons in Sottas et. al [2] extend to our approach in this scenario, and is expected to have improved performance when (as often will be the case) within subject variability varys from individual to individual. This latter scenario was investigated in the simulations (*r*_1_ ≠ 0), where it was shown that the fully Bayesian model and approximate EM model that accommodate differing within subject variability are an improvement on the standard (static) reference range.

Furthermore, a time-efficient alternative of the Bayesian approach was added to the existing literature using an approximate EM algorithm in which the generation of dynamic reference ranges relies on a few sufficient statistics at the individual level that contain all the required information from previous observations.

The findings from the simulation study presented show that the Bayesian approach was the best method for developing reference ranges under all scenarios considered and can be used effectively to detect abnormal measurements in longitudinal monitoring of an individual biomarker. However, it has also been shown that the Bayesian approach can be replaced with the static reference ranges when the WSV is considerably larger than BSV (i.e., when *r*_2_ is increasing).

The approximate EM algorithm was found to be the most efficient approach, in terms of computational cost, in developing reference ranges when the number of observations is large. Despite the minor difference between the approximate EM algorithm and the Bayesian approach in terms of performance, the former outperforms the latter in terms of processing time.

The benefits of using dynamic approaches to create individualised reference ranges were also highlighted by implementing the methods on real biomarker data collected longitudinally from diabetic patients and elite athletes. An interactive web based application was also developed to generate static and dynamic reference ranges for a variety of applications. The application is accessible at https://pci-nuig.shinyapps.io/barshinydemo/.

## 7 Conclusion

In this study, methods for developing personalized adaptive reference ranges for longitudinally recorded clinical biomarkers were presented and compared with an intention to help researchers and physicians to make more reliable decisions in terms of what can be considered as the normal physiology of an individual. The approaches undertaken included a fully Bayesian model and an Empirical Bayesian counterpart estimated via a streaming EM algorithm. In the former approach, the reference ranges were estimated from the posterior predictive distribution while in the latter the generation of reference ranges only rely on a few summary statistics that contain all the required information from previous observations. The results have shown that the proposed adaptive methods are capable of triggering ‘alerts’ and can be used as an early warning system that warrant further attention and review. The methods developed will have a broader appeal as they can be applied to any domain that involves sequential monitoring of individuals (medical devices, disease diagnosis) or processes (e.g. quality control). Further work will include incorporating covariates into the models as well as constructing multivariate dynamic reference regions when more than one biomarker is of interest.

## Supporting information

S1 Raw data (CSV)
